# How to Avoid and Handle Problems in the Placement of Cement-Augmented Fenestrated Percutaneous Pedicle Screws?

**DOI:** 10.1227/neuprac.0000000000000106

**Published:** 2024-08-28

**Authors:** Fernando Padilla-Lichtenberger, Federico Landriel, Alfredo Guiroy, Miguel Casimiro, Álvaro Silva, Santiago Hem

**Affiliations:** * Servicio de Neurocirugía, Sección Patología Raquimedular, Hospital Italiano de Buenos Aires, Buenos Aires, Argentina;; ‡ Current affiliation: Department of Neurosurgery, Hospital Italiano de Buenos Aires, Buenos Aires, Argentina;; § Clínica de Cuyo, Servicio de Cirugía de Columna, Mendoza, Argentina;; ‖ Department of Neurosurgery, Hospital da Luz, Lisbon, Portugal;; ¶ Department of Neurosurgery, Hospital da Luz-Clínica de Oeiras, Oeiras, Portugal;; ** Facultad de Medicina, Clínica Alemana Universidad del Desarrollo, Santiago, Chile

**Keywords:** Fenestrated pedicle screws, Cement augmentation, Vertebral fracture, Osteoporosis, Metastatic spine disease, Screw loosening, Pullout

## Abstract

**BACKGROUND AND OBJECTIVES:**

Pedicle screws with a central cannula and fenestrations allow cement augmentation, providing lower risk for screw loosening and pullout, especially in these patients with poor bone quality. This study aims to offer suggestions for resolving issues and reducing complications associated with the use of cement-augmented fenestrated pedicle screws.

**METHODS:**

A retrospective study was conducted across multiple centers on patients who received fenestrated pedicle screws with cement augmentation (CAFPS). Using 2-dimensional fluoroscopy guidance, we placed over 800 screws in 137 patients. Based on our analysis of common challenges and complications, 10 tips were compiled, that we believe are crucial for successfully implementing this technique, regardless of the brand or instrument used.

**RESULTS:**

The 10 tips included the following: (1) Indications of cement-augmented fenestrated pedicle screws; (2) use the K-wire blunt end in osteoporotic vertebrae; (3) know the longitude and diameter of the screw, by the measurement of the vertebrae to treat; (4) do not go bicortical; (5) clean the way of the screws fenestrae with saline; (6) protecting screw extensors with gauze; (7) measuring time and volume; (8) gently and smoothly introduce the cement; (9) do not panic. The presence of cement in the posterosuperior area adjacent to the pedicle does not necessarily indicate a leakage into the canal; and (10) fenestrated screw removal.

**CONCLUSION:**

The implementation of these tips could enhance technique performance and minimize complications in cement-augmented fenestrated pedicle screw placement.

**ABBREVIATIONS:**
CAFPS: cement-augmented fenestrated pedicle screws
HU: Hounsfield unit
PMMA: polymethylmethacrylate
VB: vertebral body.

Pathologically fragile bones are incapable to properly bear weight beyond the physiological load and suffer from fractures with minimal trauma.^1^ Poor bone quality, as a consequence of oncological processes or osteoporosis, can compromise the stability of a fusion surgery.^2-4^ A major complication is the mechanical failure caused by screw loosening.^5^ Because of the loss of trabecular architecture, the bone becomes weak, and the mechanical purchase of the pedicle screws decreases. Multiple cyclic loading prevents integration at screw-bone interface, resulting in screw loosening and pullout.^1,6^ These complications lead to worse outcomes, increased reoperation rates, longer hospital stay, and rises in health care costs.^7^ Many solutions have been proposed to reduce this risk, including expandable screws, hydroxyapatite-coated screws, bicortical screw purchase, larger diameter screws, and polymethylmethacrylate (PMMA) augmentation.^1,3,5,8^ Pedicle screws with a central cannula and fenestrations allow cement augmentation, providing lower risk for screw loosening and pullout, especially in these patients with poor bone quality.^5,9^ Biomechanical studies have demonstrated an increased resistance of the screw-bone interface with this technique.^4,10^

Although this method has proven to be superior over noncemented instrumentation in these patients, it also presents new technical challenges and specific complications, such as intraoperative mispositioning of the pedicle screw insertion point, postoperative screw migration, cement leakage, and risk of embolism.^2-4,8-11^. The ideal cement volume for secure screw fixation remains a topic of debate because of possible differences in spinal segments, bone conditions, vertebral body (VB) sizes, types of pedicle screws, and cement viscosity.^10^

The aim of this study was to provide recommendations based on our experience in minimally invasive spine surgery to improve the technique related to the placement of cement-augmented fenestrated pedicle screws (CAFPS).

## METHODS

A multicenter retrospective study was conducted, including 137 patients who received fenestrated pedicle screws with cement augmentation between 2016 and 2022 at 4 hospitals in Argentina, Chile, and Portugal. Over 800 screws were placed using 2-dimensional fluoroscopy guidance. After analyzing common challenges and complications, we have developed 10 tips that we believe are essential for successful implementation of this technique. The tips we describe were discussed in multiple asynchronous sessions and unanimously agreed upon. The tips are not specific to any brand or instrument. Approval from an institutional review board/ethics committee and patient consent were not necessary for this retrospective study.

### Protected Health Information Consent

The participants and any identifiable individuals consented to publication of his/her image.

## RESULTS

### Recommendations

#### Tip 1: Good Indication, Good Outcomes

CAFPS are primarily used to alleviate pain caused by osteoporotic vertebral fractures in elderly and postmenopausal women, as well as secondary osteoporosis resulting from prolonged exposure to corticosteroids, neoplasms, alcoholism, prolonged immobilization, rheumatoid arthritis, and other conditions. They also described in treating Kummell lesion concomitant vertebral fractures, neurological deficits resulting from vertebral fractures, progressive kyphosis, reinforcement of shot fixation, and pseudoarthrosis caused by vertebral fractures. When dealing with patients with poor bone quality, there is a greater risk of conventional transpedicular screws being pulled out. One of the primary benefits of augmented screws is their enhanced grip strength.

**FIGURE 1.**
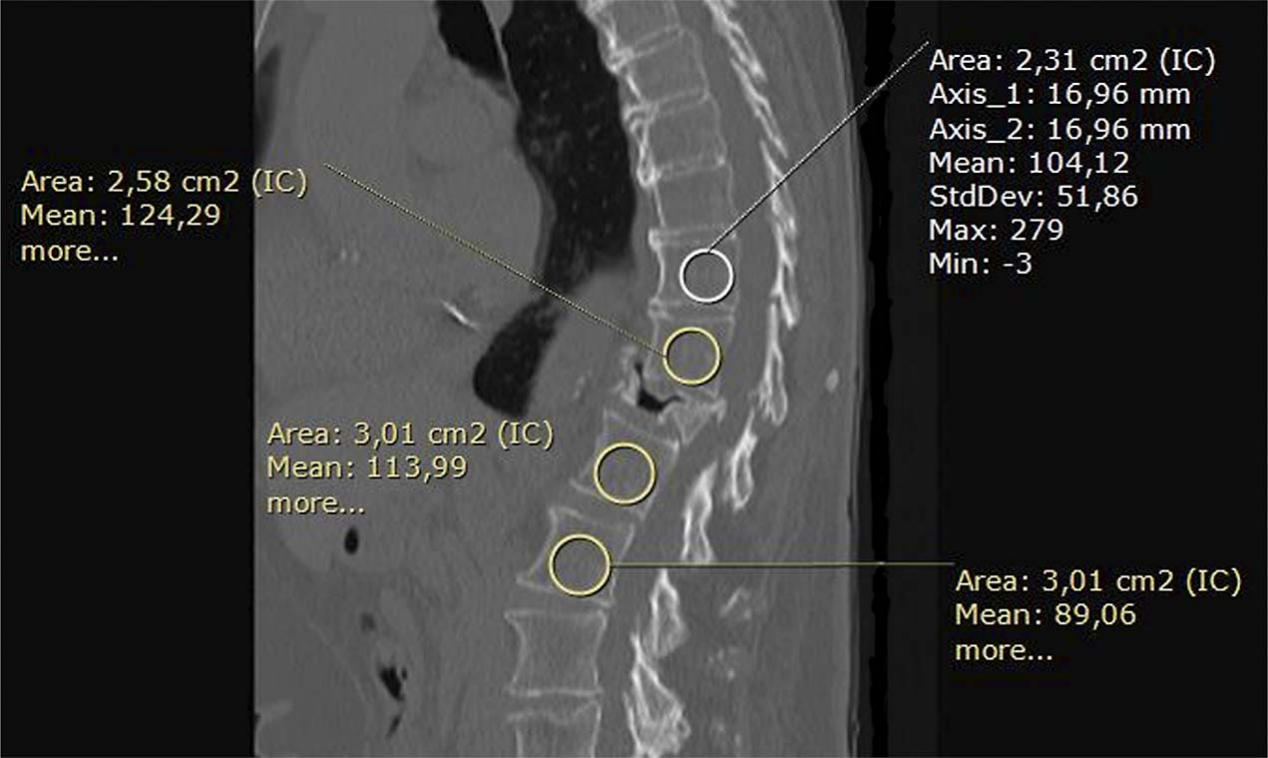
The sagittal view of the computed tomography shows the mean Hounsfield unit measurement of the spine levels (T10-L2) planned to be included in an instrumentation because of an osteoporotic fracture of T12.

Dual-energy x-ray absorptiometry remains the gold standard assessing bone mineral density (BMD), but Hounsfield units (HUs) in spinal computed tomography have emerged as a powerful complement to dual-energy x-ray absorptiometry.^12-14^ Normal BMD, with a T score ≥−1.0, corresponded to 151.7 to 184.1 HU; osteopenia, with a T score between <−1.0 or >−2.5 equals 98.5 to 120.9 HU; and osteoporosis, with a *T* score ≤−2.5 correlates to 60.5 to 100.3 HU (10). The HU can be accurately accessed, using a simple and practical method. This involves taking a sagittal view of the levels where we intend to place the screws and measuring the HU along the trajectory of the screws (Figure 1). It is recommended to perform augmentation for HU values lower than 120 HU.^15^ Augmentation increases the pullout strength by 250%.^16^ We mainly use the HU and augmentation for degenerative, trauma, and oncology cases. However, the specific types of patients and pathology for which this recommendation might apply may vary.^15^

#### Tip 2: K-Wire With a Blunt End

After the percutaneous incisions, the opening of the muscular fascia, and the correct placement of the Jamshidi needle at the entry point, use a mallet to penetrate the cortical bone smoothly, followed by manual progression. Make sure to perform this step with radiological control until the needle tip reaches the union of the anterior 2/3 and posterior 1/3 parts of the VB. Our recommendation is that in osteoporotic vertebrae, or vertebrae with poor BMD, the K-wire should be placed manually with extreme care, using its blunt edge. The use of the sharp end of the K-wire may result in unintended anterior cortical penetration, which can lead to complications such as vascular injuries and cement extravasation.

Using a spindle motor to place the K-wire results in less progression control. The correct final placement of the guidewire is reaching the anterior third of the VB.^9^

#### Tip 3: Know the Screw

Before any instrumentation, it is crucial to measure the pedicle's diameter and the screws' length at each level. To ensure accuracy, the author suggests creating a table with the levels to be treated, specifying the right and left sides, and noting any details such as tumor lesions in a pedicle or any spinal cord abnormalities. This will ensure a comprehensive and detailed approach to treatment. Avoid short screws; although the cement will exit through the distal segment of the screw, it will fill the posterior half of the VB. A leak near the vertebral canal should be avoided.

**FIGURE 2.**
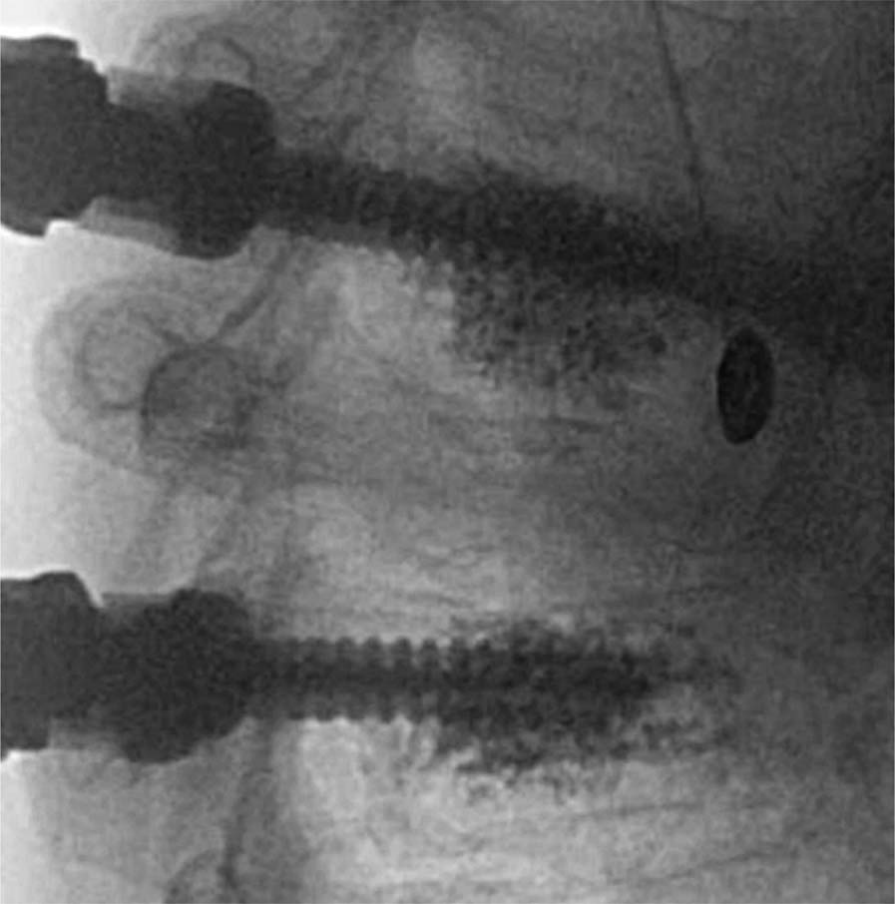
Lateral x-ray augmentation of a fenestrated screw, note the wider distribution of the polymethylmethacrylate around the vertebral body.

**FIGURE 3.**
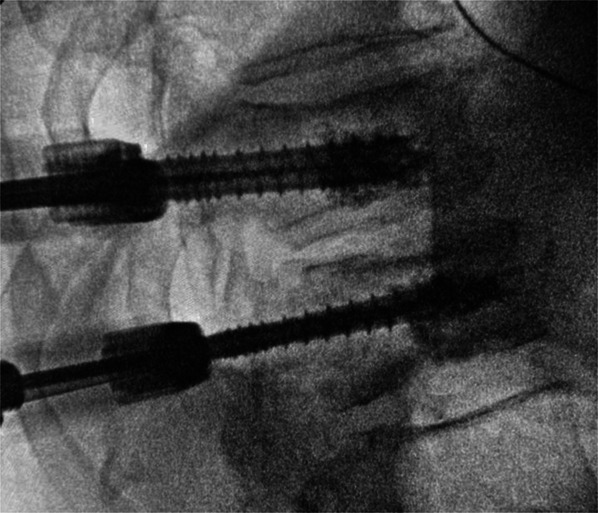
Augmentation through a cannulated screw where cement tends to fill the anterior part of the vertebral body.

The placement of fenestrated or cannulated screws is determined by the size of the pedicle. 4.5 mm cannulated screws were used for small-diameter pedicles, such as those found in high thoracic levels. We do not have any brands available in our settings that offer fenestrated screws with a diameter smaller than 5.5 mm. Fenestrated screws have a homogeneous distribution of cement around the distal part of the screw, whereas cannulated screws only inject through the tip hole, with a consequent anterior cement release (Figures 2 and 3). In solid screws, the PMMA is directly applied into the screw hole before placing it.^17^

#### Tip 4: Do Not Go Bicortical

For safe and effective screw placement, it is imperative to position the screw tip at the beginning of the anterior third of the VB. Use lateral x-ray control and maintain approximately 10 mm from the anterior cortical wall. Although the bicortical purchase has been shown to have a better grip, in these cases, breaking the anterior cortex will result in an inevitable cement leak in front of the VB with potential complications. In the case of having doubt about the integrity of the anterior cortex, you can confirm it by inserting the K-wire by its blunt end and under lateral x-ray vision feel if there is a barrier and see in x-ray if the K-wire does not exceed the limit of the anterior cortex.

#### Tip 5: Clean the Way

First, it is a crucial factor ensuring that the screw extender attachments are in line with the screws. Once the cementation cannulae are in place, we use 20 cc syringes filled with saline solution to clean the fenestrae. Often, bone fragments tend to remain in the fenestrae of the screw, making it difficult for the cement to come out later. If the blockage persists or significant pressure is required to inject the solution, reinsert the K-wire through the screw. Afterward, repeat the cleaning process in the same VB contralateral screw. The cleaning is successful when the saline solution flows out of the opposite screw of the treated vertebra (Video 1). Note that if the screw extender is badly attached to the screw, a later significant leakage of cement to the muscle space can occur. The proposed cleaning maneuver can warn of this poor connection by being able to detect a physiological solution coming out of the skin wound.

#### Tip 6: Protect the Screw Extensors

Before starting the cement augmentation process, it is advised to protect the connection between the cementation towers and screw extenders. This can be achieved by tying gauze tightly around the hole where the cement will be inserted. If any PMMA leaks during the process, the gauze can be used to clean it up. This simple maneuver is effective in preventing cement leaks from entering the wound or screw extensors, which could lead to complications when removing the tower (Video 2). A small amount of cement in the screw head, where the bar rests, is enough to induce severe difficulties in reducing and blocking the screws. If a leakage around the screw extensor is detected, we strongly recommend seeking a direct visualization control of the screw head and removing the cement before it polymerizes, ensuring that the bed of the screw head is clean. Achieving optimal vision may require a larger opening of the skin wound.

#### Tip 7: Measuring Time and Volume

**FIGURE 4.**
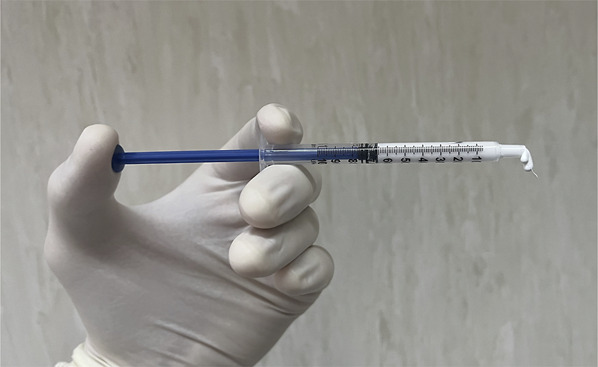
Cement directly falling because of gravity. This consistency is overly liquid and increases the leakage probability.

**FIGURE 5.**
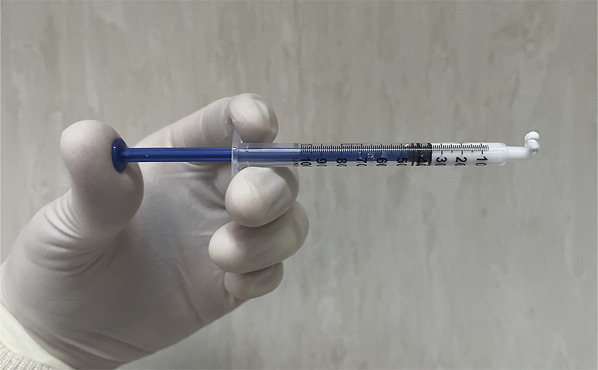
The polymethylmethacrylate optimal viscosity when a cement curl comes out upward after pushing the syringe (toothpaste consistency).

Before using cement, knowing the brand viscosity and setting time is essential. PMMA is typically prepared 7 minutes before usage. It is crucial not to rush the cement placement, as overly liquid cement can increase the risk of leakage or embolism. The optimal time to place it is usually when a cement curl comes out upward after pushing the syringe (toothpaste consistency), and it does not fall directly because of gravity or when it becomes bright (Figures 4 and 5). Before proceeding, it is crucial to calculate the necessary amount of cement. Each dose of PMMA is sufficient for 4 cemented screws, with an average of 3 to 6 cc of cement required per screw; this dosage can vary based on the size and level of the vertebra that is being cemented. Using a slightly more significant amount of cement may be appropriate when using fenestrated screws because of the more even distribution of cement in the VB. When using cannulated screws, the cement tends first to fill the front part of the VB, requiring a less solid and very synchronized application to achieve a uniform augmentation.

#### Tip 8: Gently and Smoothly

Once cement has acquired a firmer, toothpaste consistency, place 2 cc to fill the cannula, without x-ray control. An additional 1.5 to 2 cc is then placed gently and smoothly under anteroposterior and lateral fluoroscopic control. We suggest using 1 mL insulin syringes for this purpose. Finally, we use bone filler to lower the remaining cement in the cannula in the same manner (Video 2). Four screws can be augmented appropriately with a single dose of cement. Under direct lateral x-ray view, we suggest filling the cannulas sequentially by injecting 1/4 of their volume screw by screw. This allows us to observe the cement distribution and prevent any escape.

#### Tip 9: Do Not Panic!

During the process of screw augmentation, a lateral x-ray is usually employed to monitor the procedure as cement leakage behind the VB can be extremely dangerous. However, the anteroposterior x-ray control should also be used, as a posterior leakage may be present inside the canal or paraspinal area, which can only be detected through this projection. The presence of cement in the posterosuperior area adjacent to the pedicle does not necessarily indicate a leakage into the canal. In patients with poor bone quality, or after using the tap, the cement may follow the path of the screw and fill the pedicle instead (Video 3). If a small volume canal leak is detected, an anesthesiologic-induced Valsalva maneuver can allow a remodeling of the cement against the VB surface.

#### Tip 10: Fenestrated Screw Removal

The popular opinion between spinal surgeons is that removing a cemented fenestrated screw is challenging. However, this is not always the case. Over time, a fibrous layer forms between the cement and screw, preventing them from adhering. When attempting to unscrew the CAFPS, the only thing holding it in place are the cement connections that pass through the fenestrations to the bone. These connections typically break when applying the initial torque to unscrew.

Choma et al^18^ report an average extraction torque for solid augmented screws of 1.167 Nm, whereas full-fenestrated screws and partially fenestrated screws had values of 1.764 Nm and 1.794 Nm, respectively. However, these differences were not significant.

## DISCUSSION

In the context of poor BMD, several techniques were proposed to optimize pullout strength and improve the fixation, such as expandable screws, hydroxyapatite-coated screws, bicortical screw purchase, larger diameter screws, and PMMA augmentation. The placement of CAFPS stands as a true advancement within the realm of minimally invasive surgery.

### Complications

A recent meta-analysis that included 1277 patients stated that screw-related complications, such as loosening, breakage, and migration, are very low in spinal instrumentation using CAFPS for osteoporotic patients. Although cement leakage is possible, it rarely causes symptomatic neurovascular or pulmonary complications.^19^ This article of Zhang et al reported screw loosening of 2.0%; screw breakage of 0.6%; screw migration of 0.2%; cement leakage (21.8%; 1.2% symptomatic); pulmonary embolism (3.0% overall incidence and 0.8% symptomatic incidence); neurovascular complications (1.6% pooled risk); adjacent compression fracture (3.3% pooled risk); and infectious complications (3.1% pooled risk) In our series, of the 137 patients, our complication rates were similar to those described in the meta-analysis. We reported 2 cases of pulmonary embolism (1.46%); both cases remained asymptomatic; 17 cases of cement leakage (12.4%); and 3 cases of superficial infection (2.2%). No screw loosening, breakage, or migration was evidenced.

There are specific situations where fenestrated pedicle screws with cement augmentation are recommended: Patients with metastatic spinal disease who have osteopenia or osteoporosis and are unable to undergo timely treatment for osteoporosis. This is particularly beneficial for those with lytic lesions^20^; osteoporotic patients with pathological fractures that have partially responded to treatment and have a BMD outside of normal parameters; patients whose surgeon identifies a high risk of pullout during surgery because of poor BMD, Kummell lesion, or revision for loosened thick diameter screws.

However, these strong fixations can lead to other concerns, such as proximal junction kyphosis in long constructs and rapid degeneration of the adjacent level. To address these concerns, solutions such as sublaminar hooks, sublaminar wiring, or banding have been proposed.^21^

Screw augmentation increases the pullout strength by 250%.^16^ Some studies found that solid cemented pedicle screws have higher resistance to pullout than fenestrated cemented screws in biomechanical tests on artificial bone models simulating osteoporosis.^22,23^ In contrast, Choma et al^18^ found significantly higher pullout forces for the partially fenestrated than for the solid cemented screws on human osteoporotic thoracic and lumbar specimens. Conversely, Becker et al^24^ on the other hand, reported no significant difference between the solid and fenestrated cemented screws. These conflicting results might be partially explained by the different screw designs. Chen et al^25^ demonstrated that pullout force increases with the number of output holes in fenestrated screws, which could explain the poor results reported for the fenestrated screws in the studies that used screws with only one or two holes.^22,23^ By contrast, Choma et al^18^ were using 9 hole screw type and Becker et al^24^ were testing screws with 4 holes. Another possible explanation is the varying bone conditions used in the studies. Chen et al^22^ and Chang et al^23^ used artificial bone, which may not be comparable with human bone. Choma et al^18^ used highly osteoporotic bone, but without randomization.

Becker et al^24^ conducted a study on the pullout forces according to BMD, whereas Leichtle et al^10^ performed paired comparisons using 2 screw types on the pedicles of human vertebrae. Both groups had the same average BMD, accounting for natural variations. The results indicated no significant difference in pullout force between solid cemented and fenestrated cemented screws when tested on human vertebrae, taking BMD into account.

Poor BMD is a challenging situation for patients that undergo spinal fixation surgery. Despite potential challenges, CAFPS has promising outcomes in reducing screw pullout, being a valid and safe treatment option. It's the authors’ opinion, however, that the use of the high number of holes, fenestrated cemented screws, results in a simpler, shorter procedure, with less vertebral and pedicle manipulation which is a major advantage in these patients with poor bone quality.

We hope these tips can help reduce mistakes and make the learning curve shorter, safer, and less demanding.

### Limitations

This study offers valuable insights and recommendations for enhancing the technique of using CAFPS. However, several limitations must be considered:

#### Retrospective Design

The study employs a retrospective design utilizing data from multiple centers, which may introduce selection bias and limit the ability to control for confounding variables. As a retrospective analysis, the study does not incorporate randomization of subjects or controls, thereby constraining the ability to make causal inferences regarding the effectiveness of the recommendations compared to other methods.

#### Focus on a Single Technique

The study primarily addresses the use of cement-augmented fenestrated pedicle screws and does not include a comparative analysis with alternative screw augmentation techniques or non-augmented screws. This limitation restricts the scope of understanding regarding the relative effectiveness and complications associated with CAFPS.

#### Variability in Surgical Practices

The recommendations are derived from the authors' clinical experience and may not account for variability in surgical practices, screw brands, or instrumentation used across different centers. This variability could affect the generalizability of the recommendations.

#### Limited Outcome Measures

The study focuses on procedural tips and complications but lacks comprehensive outcome measures.

#### Potential for Bias

The authors' extensive experience in the field may introduce a potential bias towards endorsing the techniques they use. Independent validation from external research groups would enhance the credibility and applicability of the recommendations. These limitations should be considered when interpreting the study's findings and applying the recommendations to clinical practice.

Future research employing prospective, randomized controlled trials and broader outcome assessments would be beneficial for validating and refining these recommendations.

## CONCLUSION

The implementation of these tips could enhance technique performance and minimize complications in cement-augmented fenestrated pedicle screw placement. Although CAFPS offer enhanced grip strength and stability, they also present unique technical challenges and potential complications. However, with adherence to our recommended tips, surgeons can navigate these challenges effectively and achieve favorable outcomes for their patients. The take-home message from our study is clear: Meticulous attention to detail is paramount when using CAFPS in patients with poor bone quality. From meticulous preoperative planning, including assessment of BMD using HUs, to precise intraoperative techniques such as careful K-wire placement and judicious cement volume control, each step contributes to the overall success of the procedure.
